# The effect of values education on students' attitudes toward values: A meta-analysis of graduate theses conducted in Türkiye

**DOI:** 10.3389/fpsyg.2026.1897199

**Published:** 2026-09-03

**Authors:** Ahmet Akif Erbaş, Elif Sena Kiraz Karabulut

**Affiliations:** 1 Department of Child Care and Youth Services, Ahmet Erdoğan Vocational School of Health Services, Zonguldak Bülent Ecevit University, Zonguldak, Türkiye; 2 Ministry of National Education, Zonguldak, Türkiye

**Keywords:** attitude, education, meta-analysis, students, values education

## Abstract

The relationship between an attitude and values is a key factor in determining the strength of that attitude. Holding a value-based attitude lends strength to that attitude. The process of acquiring values begins in the family and continues systematically in schools. The literature contains a gap regarding the processes of instilling values, highlighting the need for comprehensive and up-to-date meta-analyses, while further research is needed to explain the relationship between attitudes and values across different cultures. In this research, which uses a meta-analytic approach, the effect of values education practices on students' attitudes toward values was examined. Within the scope of the study, 6,556 theses conducted in Türkiye were examined, and 62 theses were included in the meta-analysis. In accordance with the inclusion criteria, the studies in the sample represent a total of 1,788 students from preschool to university. It was determined that the implemented values education practices had a large positive effect on students' attitudes (*g* = 1.546, 95% CI = [1.304–1.788]). It was understood that values education practices conducted at different educational levels had different effects on students' attitudes. The highest effect size was found at the primary school, preschool, middle school, and high school levels, respectively. These findings provide evidence supporting the effectiveness of values education practices and offer implications for curriculum development and educational policy.

## 1 Introduction

A value system is defined as a stable organization of beliefs that influences an individual's perception of objects and events, as well as their preferences and choices (Kaymakcan and Meydan, 2011). The concept of value refers to the set of shared beliefs and judgments that determine the behavior of an individual and the community to which they belong in the face of an event or situation (Direkçi et al., 2020). Individuals are not born with values; rather, they acquire them through teaching, learning, and socialization processes. Throughout life, perceptions of values are formed through environmental factors (Yılmaz, 2006; Yeşil and Aydın, 2007). For this reason, the acquisition and internalization of values is in itself a subject and problem of education (Ulusoy and Dilmaç, 2015). The aim of the education system and schools is not only to ensure cognitive development. It also aims to support affective development and the value system of individuals (Durlak et al., 2011). The purpose of values education is to reveal the innate affective characteristics of individuals and to enable them to develop a personality committed to values (Yeşil and Aydın, 2007; Kızıler and Canikli, 2013). Regardless of the era, people strive to provide the best education (Yaman, 2012). The aim of education is not only to impart knowledge but also to shape the behavior and character of individuals. During the educational process, individuals are expected to internalize the values of the society in which they live (Ulusoy and Dilmaç, 2015). In the context, value system should be constructed, taking into account the needs and requirements of our time (Özkan, 2008). Educational institutions are one of the important structures that enable students to internalize values and shape their attitudes (Yaman, 2012). Recent international research also emphasizes that values education should not be viewed solely as a curriculum component but as a collaborative educational process involving teachers, families, schools, and the wider community. Teachers play a pivotal role in creating inclusive, dialogic, and ethically responsive learning environments where students can develop positive attitudes toward values and responsible citizenship. Accordingly, teacher education is considered a key prerequisite for the successful implementation of values education (Morgado et al., 2024).

One of the aims of research on values is to determine the orientations of value perceptions in people and to reveal the relationships between attitudes and behaviors (Mehmedoğlu and Mehmedoğlu, 2006). Understanding values is important for understanding human behavior and attitudes (Jancius and Gavenauskas, 2022). Attitude can be defined as a relatively enduring set of beliefs about an object that encourages people to behave toward that object in certain ways. Attitudes are actions that are not observable themselves but are assumed to lead to certain observable behaviors (Kağıtçıbaşı, 1976).

## 2 Value-attitude relationship

Attitudes are generalized value judgments attributed to other individuals or groups (Yang et al., 2026). Values influence attitudes, and attitudes influence behavior (Kelly, 2020; Blankenship et al., 2022). The relationship between values and attitudes is primarily based on the value-expressive function of attitudes (Aragao and Alfinito, 2021). Expressing values is an important characteristic that shows the quality of this relationship (Kristiansen and Zanna, 1991). Attitude refers to an individual's tendency to respond, events, and inanimate objects (Turkish Language Association Dictionary, 2025). An attitude is the tendency attributed to an individual that systematically shapes their thoughts, feelings, and behaviors regarding a psychological object (Kağıtçıbaşı, 1976). In social life, social values and attitudes interact reciprocally (Kasapoğlu, 1991). There is a high-level relationship between social values and life skills (Bolat and Korkmaz, 2021).

The foundations of individuals' values, attitudes, and behaviors are laid within the family. In daily life, children observe and model the attitudes and behaviors of their parents (Essiz and Mandrik, 2022). Later, values and attitudes are shaped within social life. However, negative attitudes and behaviors reflected by parents, social environment, media, etc., have a negative impact on children's value judgments and attitudes (Jancius and Gavenauskas, 2022). These negative effects have led to consequences such as increased propensity for violence, addiction, loneliness, social decay, and weakening of social cohesion in recent years, causing a disruption in the balance of values and attitudes (Dincel, 2023). In this context, many values education programs have been initiated worldwide. The Living Values Education Programme, supported by UNESCO, is one of these comprehensively prepared values education programs. In many countries where the program has been implemented, students' positive social skills have improved. Furthermore, positive changes in students' attitudes have also been reported (UNESCO, 2005).

## 3 Values education in Türkiye

Values education in Türkiye has developed and changed in parallel with values education worldwide. Since 2004, greater emphasis has been placed to values education in curricula and course content. The first official circular on values education, published in 2010, increased awareness of the subject (Ministry of National Education, 2010a). At the 18th National Education Council, recommendations were made regarding values education, the development of teacher competencies, and the production of educational materials (Ministry of National Education, 2010b). With the curriculum reforms of 2017–2018, values education became one of the central components of the curricula (Ministry of National Education, 2017). The programs identified core values such as justice, friendship, honesty, self-control, patience, respect, love, responsibility, patriotism, and helpfulness, and aimed to integrate these values into all curricula in a holistic manner (Meydan, 2019).

A new model was adopted with the curriculum change in 2024. This model emphasizes the importance of values education in the education system. Values education is at the heart of the educational programs. This approach aims to cultivate conscious, virtuous, and socially responsible individuals by developing not only their academic achievement but also their moral and social responsibilities. Within this model, values education is one of the key elements in improving the quality and effectiveness of the education system (Ministry of National Education, 2024).

## 4 Theoretical framework

Including values education in curricula and ensuring effective educational practices are crucial for both individuals and society (Çelikkaya et al., 2014). Values education practices are effective in promoting students' acquisition of values and positive behaviors (Balcı, 2008; Demirhan İşcan, 2007; Kunduroğlu, 2010). Intervention studies aimed at values and character education positively influence students' behaviors and attitudes (Jeynes, 2017). There literature also includes studies examining the effectiveness of values education practices (Bulach, 2002; Karma and Kahil, 2005; Keskinoğlu, 2008; Aladağ, 2009; Neslitürk, 2013; Uzunkol, 2014) and that examine studies on values education from a metasynthetic perspective (Elbir and Bağcı, 2013; Budak et al., 2023; Dincel, 2023; Yazar and Kaya, 2023).

There are also meta-analysis studies on values education. Findings from previous meta-analyses indicate that it has been determined that moral education has a significant effect on moral judgment (Schlaefli et al., 1985), there is a low level of interaction between personality traits and values (Parks-Leduc et al., 2014), values education practices yield successful results (Tulunay Ateş, 2017), character education intervention studies have positive (Jeynes, 2017) and low-level (Brown et al., 2022) effects on students' behavior, and the drama method has a large effect in instilling values in students (Yıldırım, 2022). One of the comprehensive studies conducted is the study titled “What is the Use of Character Education?” (Berkowitz and Bier, 2007). As a result of the meta-analysis conducted on the data of this study, it was determined that character education intervention studies have a small level of effect (Johnson et al., 2022). According to Bandura's social learning theory, individuals acquire behaviors, attitudes, and values through observation, modeling, and social reinforcement. In this context, it is an expected outcome that values education practices will influence students' attitudes.

As can be observed, previous meta-analyses have primarily focused on character education interventions (Jeynes, 2017; Johnson et al., 2022), moral education (Schlaefli et al., 1985), the relationship between values and personality (Parks-Leduc et al., 2014), or the effectiveness of specific instructional approaches, such as drama-based values education (Yıldırım, 2022). Although these studies provide valuable evidence, they do not specifically synthesize the effects of values education practices on students' attitudes toward values within the Turkish educational context. Moreover, to the best of our knowledge, no previous meta-analysis has systematically synthesized the findings of experimental master's theses conducted in Türkiye, despite the fact that graduate theses constitute a substantial body of intervention research in the field. Therefore, an important gap remains regarding the overall effectiveness of values education practices on students' attitudes. The present study addresses this gap by providing a comprehensive meta-analysis of experimental studies conducted in Türkiye and by examining whether the effectiveness of values education differs according to educational level, curriculum period, and thesis type. In this respect, the study makes an original contribution to the literature by offering context-specific evidence that can inform future research, educational policy, and classroom practice.

The aim of this research is to determine the magnitude of the effect of values education practices on student attitudes, according to the meta-analysis results of studies in this field. In this context, the following questions were addressed:

1. What is the overall effect size of values education practices in Türkiye on students' attitudes toward values?
2. Does the effect of values education practices on students' attitudes toward values differ significantly according to educational level (preschool, primary school, secondary school, high school, university)?
3. Does the effect of values education practices on students' attitudes toward values differ significantly according to the curriculum implemented in schools (before 2018, after 2018)?
4. Does the effect of values education practices on students' attitudes toward values differ significantly according to the thesis type?

## 5 Method

**Figure 1:**
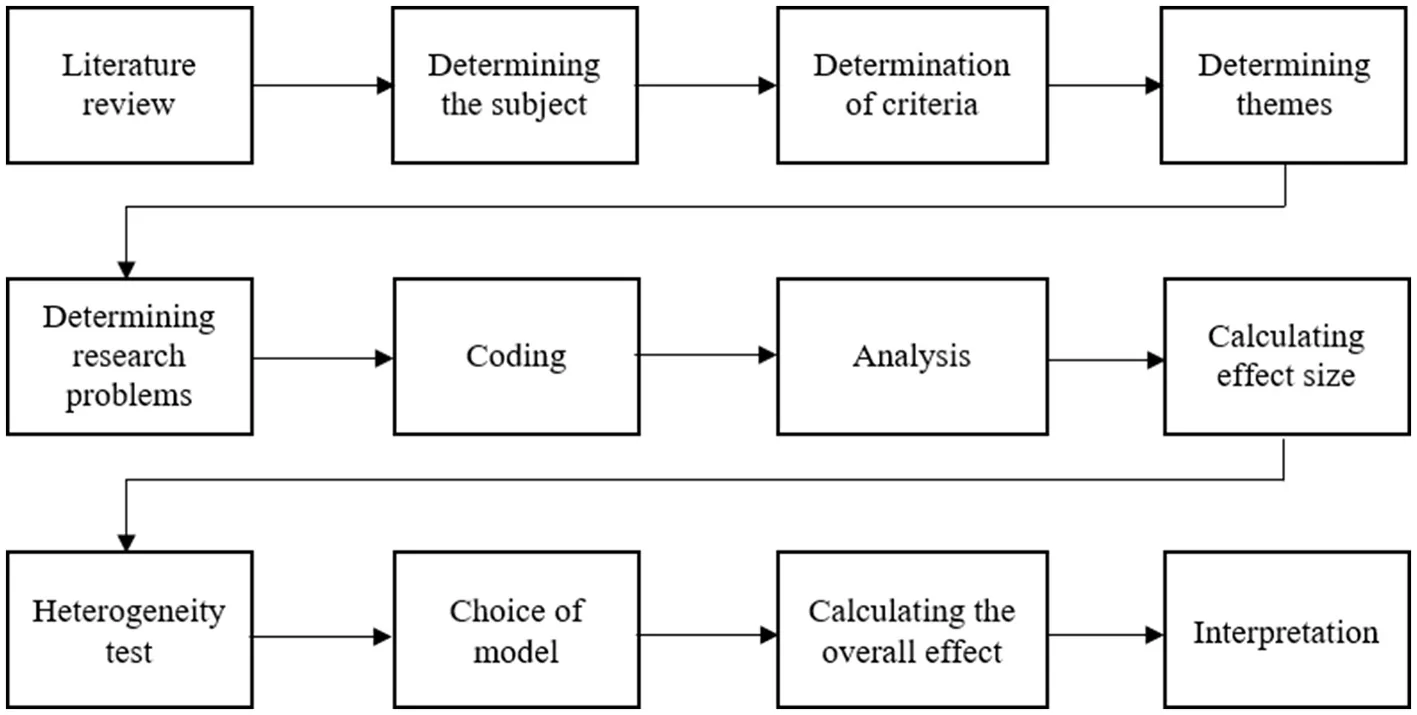
Meta-analysis process.

In our study, the meta-analysis method, one of the literature review techniques, was used to provide a comprehensive synthesis of values education practices. Standard guidelines for high-quality systematic reviews and meta-analyses were followed (Alexander, 2020; Pigott and Polanin, 2019). The meta-analysis process is illustrated in Figure 1 (Dinçer, 2021).

### 5.1 Inclusion/exclusion criteria

To determine which studies would be included and excluded from our research, specific criteria were established. These criteria are as follows:

1. The studies included in the meta-analysis must be master's and doctoral theses conducted and published in Türkiye. In Türkiye, thesis processes are monitored by the student, advisor, and thesis monitoring committee (3 people) and go through jury approval (3–5 people). This criterion is to reduce the risk of publication bias. Furthermore, a significant portion of experimental studies on values education in Türkiye have been conducted within the scope of postgraduate theses.
2. No lower publication year limit was applied. The search included all eligible theses completed up to December 31, 2025. Data search via keywords reveals that the first thesis on values education was completed in 1978. The first thesis included in the study was completed in 1999, and the last thesis in 2025.
3. The studies should include pre-tests and post-tests administered to both experimental and control groups. The experimental group should have received specific values education after the pre-test. The control group should not have received specific values education.
4. The studies included in the meta-analysis must have appropriate data. The sample sizes, pre-test, post-test, standard deviation, and mean scores of both the experimental and control groups should be provided.
5. The sample of the study included in the meta-analysis should consist of preschool, primary, secondary, high school, and university students.

**Figure 2:**
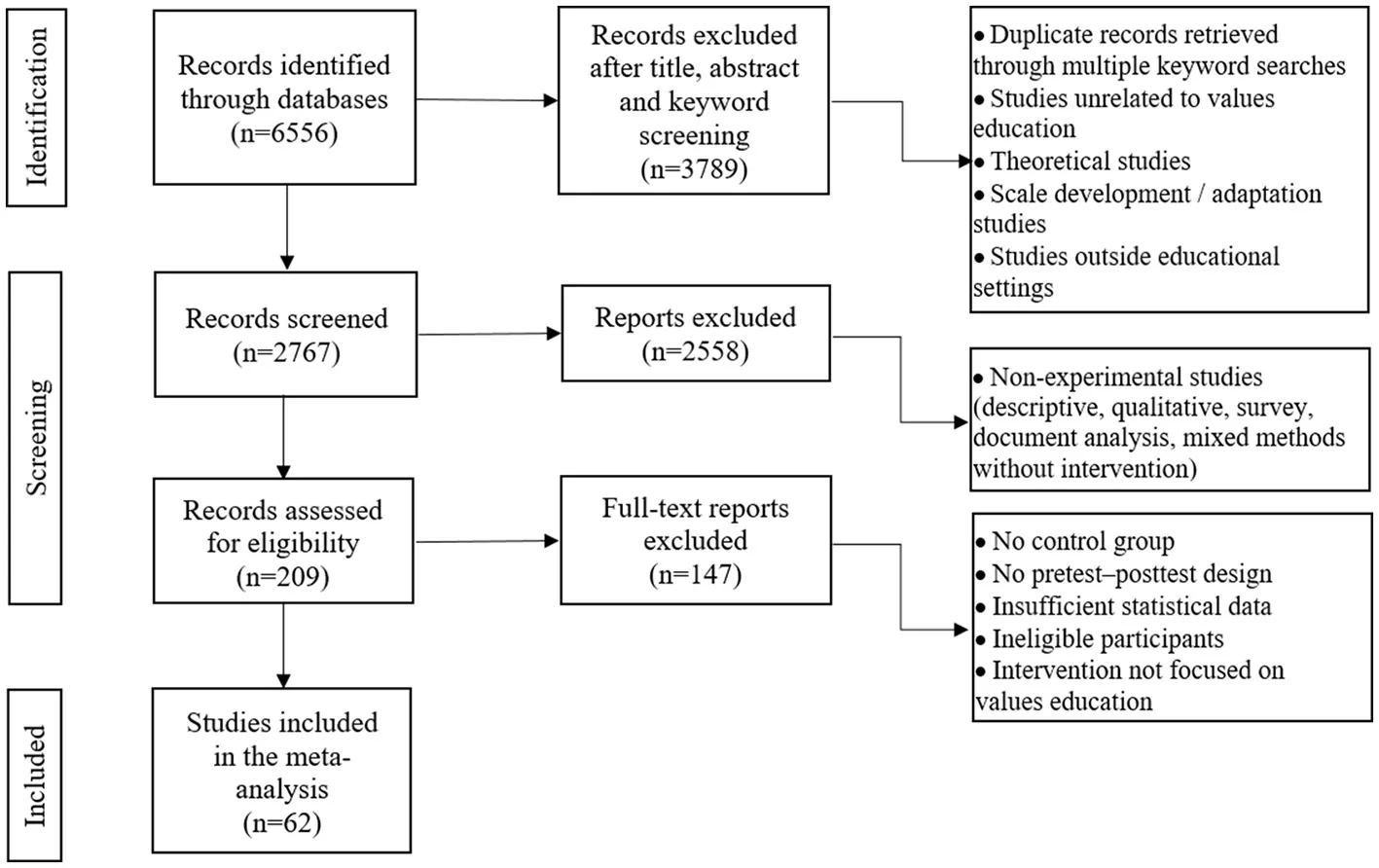
The PRISMA flowchart illustrates the selection of included studies.

The PRISMA flowchart for the selection and inclusion of studies is presented in Figure 2 (Page et al., 2021).

### 5.2 Literature search and study selection

The literature search was conducted in the Council of Higher Education (CoHE) National Thesis Center (YÖK National Thesis Center), which constitutes the most comprehensive repository of graduate theses completed in Türkiye. The search included all studies available up to December 31, 2025, and no lower publication year limit was imposed. The following Turkish keywords were used individually and in combination: values, values education, moral, moral education, character education, and value acquisition. These keywords were selected to ensure comprehensive coverage of interventions related to values education. The literature search was conducted using the advanced search function of the CoHE National Thesis Center. Boolean operators (AND, OR) were applied to maximize both the sensitivity and specificity of the search. Initially, each keyword was searched individually (e.g., value, values education, moral, moral education, character education, and value acquisition). Subsequently, combinations of these keywords were searched using the OR operator to identify studies employing different terminology (e.g., “values education” OR “character education” OR “moral education”). The AND operator was then used to combine intervention-related concepts with educational contexts where necessary (e.g., “values education” AND education; “character education” AND school). Searches were conducted using Turkish equivalents of these terms because all eligible studies were master's and doctoral theses completed in Türkiye. Search fields included thesis title, abstract, and keywords. All records retrieved from different search combinations were exported and screened manually. Duplicate records identified across different keyword combinations were removed before the screening process.

The initial search yielded 6,556 records. First, titles, abstracts, and keywords were screened to remove studies unrelated to values education interventions, duplicate records identified through different keyword combinations, theoretical studies, scale development studies, and studies outside the educational context. Following this screening, 2,767 potentially relevant studies remained. In the second stage, the full texts of these studies were examined to determine whether they employed an experimental or quasi-experimental design. Studies using descriptive, qualitative, correlational, survey, document analysis, mixed-methods without an experimental intervention, or other non-experimental research designs were excluded (*n* = 2,558), leaving 209 studies for detailed eligibility assessment.

During the eligibility assessment, the remaining studies were evaluated according to the predefined inclusion criteria. Studies were excluded if they (a) did not include both experimental and control groups, (b) lacked pretest and posttest measurements, (c) did not implement a specific values education intervention in the experimental group, (d) failed to report sufficient statistical information (sample size, mean, and standard deviation) for effect size calculation, or (e) included participants outside the target educational levels (preschool to university). After applying these criteria, 62 studies met all eligibility requirements and were included in the final meta-analysis. The included studies are presented in Table 1. These studies represented a total sample of 1,788 participants. The complete identification, screening, eligibility, and inclusion process is presented in the PRISMA 2020 flow diagram (Figure 2).

**Table 1:** Studies included in the study.

References	Curriculum	Type of thesis	Education level	Experimental group (*n*)	Control group (*n*)
Dilmaç (1999)	Before 2018	Master's	Primary school	18	18
Aydın (2008)	Before 2018	Master's	Middle school	10	10
Demir (2008)	Before 2018	Master's	Middle school	10	10
Çetin Balcı (2009)	Before 2018	Master's	Primary school	72	79
Öztürk Samur (2011)	Before 2018	PhD	Pre-school	22	22
Coşkun (2012)	Before 2018	Master's	University	31	31
Karagöz (2013)	Before 2018	PhD	Primary school	20	20
Izgar (2013)	Before 2018	PhD	Middle school	20	20
Sobi (2014)	Before 2018	Master's	Middle school	25	25
Yarar Kaptan (2015)	Before 2018	PhD	Primary school	23	22
Sapsağlam (2015)	Before 2018	PhD	Pre-school	20	20
Kocabıyık (2015)	Before 2018	Master's	Middle school	24	24
Akarsu (2005)	Before 2018	PhD	Middle school	23	20
Kirmanoğlu (2016)	Before 2018	Master's	Primary school	20	20
Gümüş (2016)	Before 2018	PhD	Primary school	32	33
Ada (2016)	Before 2018	Master's	Pre-school	21	21
Taş (2016)	Before 2018	PhD	Primary school	31	31
Batti (2016)	Before 2018	Master's	Middle school	36	34
Gökalp (2017)	Before 2018	Master's	Middle school	24	24
Temel (2017)	Before 2018	Master's	Primary school	16	16
(Okumuş 2017)	Before 2018	PhD	High school	30	28
(Okumuş 2017)	Before 2018	PhD	High school	30	30
Bozkır (2018)	Before 2018	Master's	High school	30	30
Akgün (2018)	Before 2018	Master's	Pre-school	20	20
Külünkoǧlu (2018)	Before 2018	PhD	Middle school	19	23
Güneş (2018)	Before 2018	Master's	Pre-school	20	20
Coşkun (2019)	Before 2018	Master's	Middle school	90	84
Tekin (2019)	Before 2018	Master's	Primary school	19	17
Erim (2019)	Before 2018	Master's	Middle school	24	27
Yıldırım (2019)	Before 2018	PhD	Middle school	16	16
Altunsoy (2019)	Before 2018	Master's	Primary school	17	18
Karataş (2019)	After 2018	Master's	Primary school	27	27
Yılmaz (2019)	After 2018	Master's	Primary school	30	30
Gürgür (2019)	After 2018	Master's	Middle school	13	15
Özçelik (2019)	After 2018	Master's	Primary school	40	40
Kılıç (2020)	After 2018	Master's	Middle School	101	110
Dalyan (2020)	After 2018	Master's	Primary school	22	19
Veziroğlu (2020)	After 2018	Master's	Middle school	10	10
Sungur (2022)	After 2018	Master's	Middle school	15	15
Evlice (2022)	After 2018	PhD	Middle school	30	30
Balık (2022)	After 2018	Master's	Pre-school	22	22
Ata (2022)	After 2018	PhD	University	27	26
Evcimik (2023)	After 2018	PhD	Middle school	166	166
Aydın (2023)	After 2018	Master's	Middle school	20	20
Gül (2023)	After 2018	Master's	Middle school	20	20
Varış (2023)	After 2018	Master's	Middle school	25	25
Hüdavendigar (2024)	After 2018	PhD	Middle school	18	18
Hüdavendigar (2024)	After 2018	PhD	Middle school	18	18
Hüdavendigar (2024)	After 2018	PhD	Middle school	18	18
Özdemir (2024)	After 2018	PhD	High school	42	36
Masraf (2024)	After 2018	Master's	Middle school	21	21
Özses (2025)	After 2018	Master's	Middle school	12	15
Macun (2025)	After 2018	PhD	Pre-school	24	20
Akcan (2025)	After 2018	PhD	High school	49	50
Soylu (2025)	After 2018	Master's	Pre-school	22	22
Koç (2025)	After 2018	Master's	Middle school	28	27
Koç (2025)	After 2018	Master's	Middle school	28	27
(Topal 2025)	After 2018	Master's	Middle school	17	18
Madran (2025)	After 2018	PhD	High school	28	34
Tırıl (2025)	After 2018	Master's	Middle school	25	22
Tırıl (2025)	After 2018	Master's	Middle school	31	22
Aydın (2025)	After 2018	PhD	Pre-school	26	26

### 5.3 Coding reliability

The coding system's inclusion/exclusion criteria were developed based on the study bibliography and variables (curriculum, thesis type, study group, number of experimental/control groups, means, standard deviations). Some theses reported multiple independent comparisons. These effect sizes were included separately only when the participant groups and intervention conditions were independent of each other. The data were independently coded by researchers and checked several times for accuracy. The consistency of the data was then cross-validated by the researchers. Discrepancies in the data were discussed among the researchers, and then expert opinion was sought before a final decision was made. The expert was an academic specializing in meta-analysis studies. Inter-coder consistency was assessed using the model formulated by (Miles and Huberman 1994). The model suggests inter-coder consistency to be above 80%. In this study, the inter-coder agreement was found to be above 90%.

### 5.4 Data analysis

Data obtained in accordance with the inclusion criteria from the literature review were analyzed using Stata 16. Hedges' g was used to calculate the effect size. Kendall's tau and Egger's regression test were used to analyze publication bias. Heterogeneity was examined using the Cochran's Q test and I^2^ statistic. Cochran's Q test for between-group heterogeneity was used to compare the effect sizes of thesis type, curriculum, and educational level variables. The significance level was set at *p* < 0.05.

In interpreting the findings obtained from the analyses, Cohen's (1988) effect size classification was used as a reference. Effect sizes between 0.20 and 0.50 are considered small, those between 0.50 and 0.80 are considered medium, and those of 0.80 and above are classified as large.

## 6 Findings

**Figure 3:**
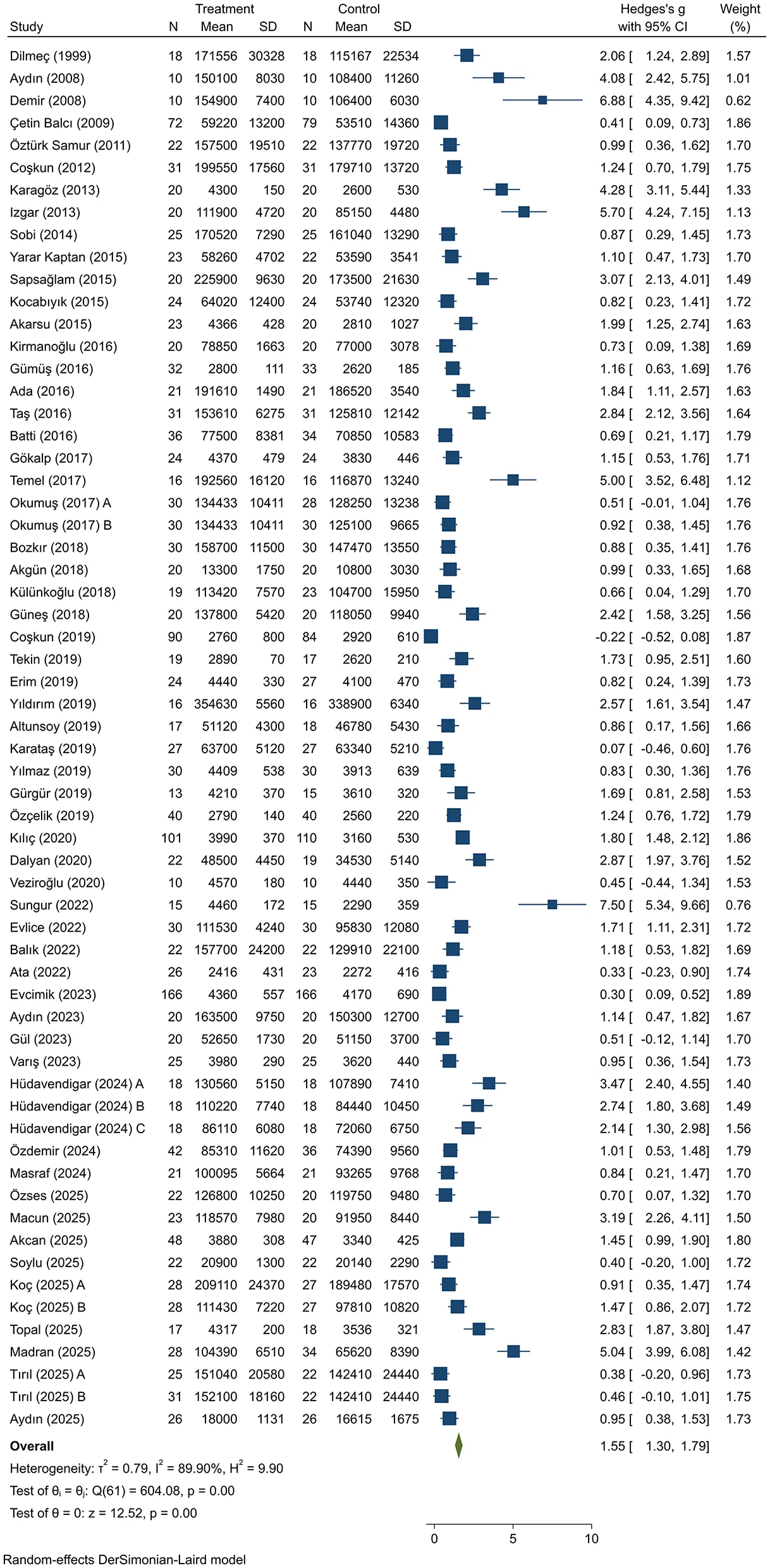
Forest plot of the studies included in the research.

The forest plot showing the effect sizes of the studies included in the research is shown in Figure 3. Almost all studies were found to have a significant effect in favor of the experimental group. Sixty-one studies yielded positive effect sizes favoring the experimental group, whereas one study (Coşkun, 2019) yielded a negative effect size favoring the control group. Overall, the majority of studies favored the experimental group.

A random effects model (Der-Simonian-Laird) was used. Hedges' g represents the standardized effect size. 95% CI: 95% confidence interval; Q: Cochran's Q statistic; I^2^: percentage heterogeneity of total variance; τ^2^: estimate of variance between groups. Qb: statistic shows the difference between subgroups. ^*^p < 0.05 is statistically significant.

Based on the inclusion criteria, 62 theses were the subject of the research. Of these theses, 39 were master's theses, and 23 were doctoral theses. Two of the studies were conducted at the university level, six at the high school level, nine at the preschool level, fourteen at the primary school level, and 31 at the middle school level. It is observed that 31 of the studies were conducted before the 2018 curriculum change, and 31 were conducted after the 2018 curriculum change (Table 2).

**Table 2:** Effect sizes and heterogeneity values.

Category	Subcategory	*n*	Hedges' g	95% CI	*p*	Q	I^2^ (%)	τ^2^
Curriculum	Before 2018	31	1.656	1.289–2.023	0.000	305.93	90.19	0.915
	After 2018	31	1.453	1.124–1.783	0.000	297.88	89.93	0.742
Type of Thesis	Master's	39	1.280	1.008–1.552	0.000	292.01	86.99	0.606
	PhD	23	1.981	1.508–2.453	0.000	303.85	92.76	1.181
Education Level	Pre-school	9	1.611	1.024–2.199	0.000	50.06	84.02	0.670
	Primary School	14	1.661	1.120–2.202	0.000	135.37	90.40	0.921
	Middle School	31	1.551	1.192–1.910	0.000	332.19	90.97	0.863
	High School	6	1.527	0.735–2.319	0.000	62.58	92.01	0.883
	University	2	0.792	−0.099–1.683	0.082	5.13	80.52	0.333
General		62	1.546	1.304–1.788	0.000	604.08	89.90	0.792
**Category**			**df**		Qb		***p***	
**Differences between groups**								
Curriculum			1		0.65		0.421	
Type of thesis			1		6.35		0.012	
Education level			4		2.95		0.566	

When heterogeneity findings were examined, high heterogeneity was detected for all studies (Q = 604.08; *p* < 0.05; I^2^ = 89.90%). Heterogeneity was found to be statistically significant and high in all subgroups related to curriculum, thesis type, and study group. The high τ^2^ values across all subgroups indicate a significant level of true variance between studies. Therefore, a random effects model was used in the analyses, and effect sizes were estimated using the DerSimonian–Laird method.

According to the Egger regression test results, the β_1_ coefficient was determined to be 7.48 (SE = 0.604; *z* = 12.40; *p* < 0.05) in the analysis examining the effects of small studies. Similarly, in the analysis performed by including the moderator variables (curriculum, thesis type, and study group) in the model, the β_1_ coefficient was found to be 7.36 (SE = 0.587; *z* = 12.53; *p* < 0.05). When the Begg test results were examined, the Kendall's score was found to be 1007.00 (SE = 164.634; *z* = 6.11; *p* < 0.05). These findings indicate that small study effects exist among the studies included in the meta-analysis, and therefore, a potential risk of the findings suggest a possible small-study effect. However, the relatively high level of heterogeneity in the study (I^2^ > 89%) suggests that the asymmetry in the funnel plot may not be solely due to publication bias. Differences in study duration, sample characteristics, teaching methods, measurement tools, and cultural context may also have contributed to small study effects.

The overall effect size for the studies was calculated as *g* = 1.546 (95% CI: 1.304–1.788). When examined on a study-by-study basis, the effect sizes were found to range from −0.223 to 7.501. The highest effect size value achieved within the scope of the study was *g* = 7.50 (Sungur, 2022), and the smallest effect size value was *g* = −0.22 (Coşkun, 2019).

According to the subgroup meta-analysis results, the effect size was calculated as *g* = 1.656 (95% CI: 1.289–2.023) in the pre-2018 group and *g* = 1.453 (95% CI: 1.124–1.783) in the post-2018 group in terms of curriculum. Across individual studies, it is seen that the effect sizes ranged from −0.223 to 6.881 in the pre-2018 group and from 0.069 to 7.501 in the post-2018 group. The subgroup difference test revealed no statistically significant difference in effect sizes according to the curriculum (Qb = 0.65; *p* = 0.421).

According to thesis type, the effect size was found to be *g* = 1.280 (95% CI: 1.008–1.552) for master's theses and *g* = 1.981 (95% CI: 1.508–2.453) for doctoral theses. At the individual study level, it was observed that the effect sizes ranged from −0.223 to 7.501 for master's theses and from 0.302 to 5.698 for doctoral theses. The difference test between subgroups revealed a statistically significant difference in effect sizes according to thesis type (Qb = 6.35; *p* = 0.012). This finding indicates that the effect sizes obtained from doctoral theses are higher than those obtained from master's theses.

According to the study groups, the combined effect size was calculated as *g* = 1.611 (95% CI: 1.024–2.199) in the preschool group, *g* = 1.661 (95% CI: 1.120–2.202) in the primary school group, *g* = 1.589 (95% CI: 1.218–1.959) in the middle school group, *g* = 1.527 (95% CI: 0.735–2.319) in the high school group, and *g* = 0.792 (95% CI: −0.099–1.683) in the university group. When examined on a study basis, it is observed that the effect sizes ranged from 0.401 to 3.188 in the preschool group, from 0.069 to 5.002 in the primary school group, from −0.223 to 7.501 in the middle school group, from 0.514 to 5.036 in the high school group, and from 0.334 to 1.243 in the university group. The results of the subgroup difference test showed no statistically significant difference in effect sizes among the study groups (Qb = 2.95; *p* = 0.566). This finding indicates that the effect sizes did not differ significantly among the study groups.

## 7 Conclusion and discussion

This meta-analysis synthesized the findings of 62 postgraduate theses conducted in Türkiye between 1999 and 2025 to determine the effectiveness of values education practices on students' attitudes toward values. The overall effect size obtained from the analysis (*g* = 1.546) indicates that values education interventions have a large, positive, and statistically significant effect on students' attitudes. According to Cohen's (1988) classification, this effect size corresponds to a large effect. The findings suggest that students who participate in values education programs develop more positive attitudes toward targeted values than those who receive traditional instruction. These findings are also consistent with recent international discussions emphasizing that teachers are central to the successful implementation of values education. Beyond transmitting knowledge, teachers are expected to foster ethical reasoning, democratic participation, and responsible citizenship through their instructional practices. Accordingly, effective teacher education and continuous professional development are essential for creating learning environments that support students' moral and social development (Morgado et al., 2024).

This finding is consistent with previous meta-analytic and experimental studies conducted in the field. For example, (Bozkurt 2023) reported a large positive effect (*g* = 1.575) of values education practices implemented in primary schools. Similarly, (Tulunay Ateş 2017) found that values education interventions had a positive and significant effect on students, while (Yıldırım 2021) reported that drama-based instructional practices produced substantial improvements in students' attitudes. Internationally, (Schlaefli et al. 1985) concluded that moral intervention programs significantly contribute to students' moral and attitudinal development. The convergence of these findings across different studies and educational settings provides strong evidence that values education is an effective educational approach for fostering positive attitudes and supporting students' moral development.

The large positive effect identified in this study can also be interpreted within contemporary theoretical perspectives concerning the relationship between values and attitudes. (Blankenship et al. 2022) argue that attitudes grounded in personal values are generally held with greater certainty and clarity, making them more resistant to change and more influential in guiding future behavior. Values education practices may therefore strengthen students' attitudes by helping them establish meaningful connections between abstract values and their personal belief systems. When students internalize values such as honesty, responsibility, respect, helpfulness, and justice, these values become part of their self-concept and contribute to the development of stronger and more enduring attitudes. Consequently, the large effect size observed in this study may reflect not only increased awareness of values but also a deeper process of value internalization.

Similarly, (Ponizovskiy et al. 2019) emphasize that values influence attitudes and behaviors through value-instantiating beliefs, which enable individuals to perceive specific behaviors as expressions of particular values. Values education activities frequently employ stories, drama, role-playing, cooperative learning, case studies, and real-life examples that help students connect abstract values with everyday actions. Such educational experiences may strengthen students' beliefs about how values are manifested in real life, thereby increasing the likelihood that they develop positive attitudes toward those values. From this perspective, the effectiveness of values education may stem from its ability to transform abstract moral concepts into concrete and meaningful experiences.

Moderator analyses revealed that values education interventions produced positive effects across all educational levels. Although the largest effect size was observed at the primary school level (*g* = 1.661), followed by preschool (*g* = 1.611), middle school (*g* = 1.551), and high school (*g* = 1.527), no statistically significant differences were found among educational levels. This finding suggests that values education can be effectively implemented throughout the educational continuum. Nevertheless, developmental characteristics may help explain why relatively higher effect sizes were observed during the early years of education.

The findings obtained for preschool education are particularly noteworthy. The effect size calculated from the nine preschool studies included in the analysis (*g* = 1.611) indicates a large positive effect. This result is consistent with Yıldırım's (2021) meta-analysis, which reported a high effect size for drama-based educational interventions at the preschool level. Early childhood is widely recognized as a critical developmental period during which socio-emotional learning, empathy, moral awareness, and prosocial behaviors rapidly develop. (Pekdoğan and Korkmaz 2017) emphasize that the preschool period plays a fundamental role in character formation and the acquisition of basic life skills. Therefore, values education interventions implemented during early childhood may exert long-term effects on children's attitudes and future behavioral tendencies.

The large effect size was observed at the primary school level (*g* = 1.661). This finding is highly consistent with the results reported by (Bozkurt 2023), who found a large positive effect size (*g* = 1.575) for values education practices in primary schools. Similarly, (Yıldırım 2021) reported a large effect size (*g* = 1.109) for drama-based instructional practices, whereas (Tulunay Ateş 2017) reported a moderate effect size (*g* = 0.715). Although the magnitude of effects varies across studies, the overall pattern consistently demonstrates that primary school is one of the most effective periods for values education interventions. During this stage, children actively construct social norms, moral judgments, and interpersonal relationships. Consequently, educational activities focusing on values may have a particularly strong influence on students' attitude formation and social development.

Values education interventions also demonstrated large positive effects at the middle school and high school levels. The effect size for middle school students (*g* = 1.551) supports the findings of (Tulunay Ateş 2017), who reported a very large effect size at this educational level. Likewise, the positive findings obtained at the high school level are partially consistent with (Jeynes 2017), who argued that character education programs may have particularly large effects among adolescents. However, adolescents' increasing autonomy, identity exploration, and sensitivity to peer influence may affect the ways in which values education programs are experienced and internalized. Therefore, values education at these levels may benefit from more reflective, participatory, and discussion-based instructional approaches.

Another important finding concerns the comparison of studies conducted before and after the 2018 curriculum reform in Türkiye. Significant revisions were introduced in the curriculum, including the explicit identification of core values such as justice, friendship, honesty, self-control, patience, respect, love, responsibility, patriotism, and helpfulness. Furthermore, the revised curriculum adopted a constructivist and skills-oriented approach that emphasizes active student participation and experiential learning (Aslan, 2021). Despite these substantial changes, no statistically significant differences were found between studies conducted before and after 2018. The effect size for studies conducted before 2018 (*g* = 1.656) and after 2018 (*g* = 1.453) remained at a similarly high level. This finding suggests that values education has maintained its effectiveness despite curricular reforms. It may also indicate that the effectiveness of values education depends not only on curriculum design but also on factors such as implementation quality, teacher competencies, classroom climate, school culture, and student engagement.

Despite the moderator analyses conducted according to curriculum period, thesis type, and educational level, considerable between-study heterogeneity remained (I^2^ = 89.90%). Therefore, the overall effect size should be interpreted with caution. Variables such as intervention duration, pedagogical approach, measurement instruments, targeted values, and school context could not be examined because they were not reported consistently across the included theses. These factors may partly explain the observed heterogeneity. Future meta-analyses based on more standardized primary studies should investigate these variables to provide a more comprehensive explanation of between-study differences.

Another issue that should be considered when interpreting the findings is the potential influence of small-study effects and publication bias. Both the Egger regression and Begg rank correlation tests indicated statistically significant asymmetry. However, these findings should be interpreted cautiously because the included studies exhibited substantial between-study heterogeneity (I^2^ = 89.90%), which itself may contribute to funnel plot asymmetry. Moreover, graduate theses differ considerably in terms of intervention duration, instructional approaches, sample characteristics, and measurement instruments, all of which may have contributed to the observed variability. Therefore, although the overall effect size remained large, it should be interpreted with appropriate caution. Future meta-analyses should further evaluate the robustness of these findings using additional sensitivity analyses, such as trim-and-fill or leave-one-out procedures.

Overall, the findings of this meta-analysis suggest that values education practices implemented in the graduate theses included in this review are associated with positive effects on students' attitudes toward values. However, these findings should be interpreted with caution because the included studies were conducted exclusively in Türkiye and substantial between-study heterogeneity was observed. Therefore, the generalizability of the findings to other educational systems and cultural contexts is limited. Nevertheless, the findings indicate that values education may represent a promising educational approach for promoting positive attitudes toward values within the Turkish educational context. Future research including studies from different countries and educational settings is needed to determine whether these findings can be generalized more broadly.

## 8 Recommendations of the study

Since this study is limited to postgraduate theses in Türkiye, comparative analyses should be conducted by including internationally conducted research. The meta-analysis focuses only on experimental studies. More comprehensive conclusions regarding values education can be reached by also evaluating qualitative research, case studies, and mixed methods studies. Longitudinal studies should be conducted to examine the long-term effects of values education practices on student attitudes. The current study addressed the effects on student attitudes. The holistic impact of values education should be evaluated by also investigating the perspectives of teachers, parents, and school administrators. Similar effect sizes were found in studies conducted before and after the 2018 curriculum change. How the curriculum changes were reflected in practice and why the expected difference did not occur should be investigated in detail. It is recommended that values education continue sustainably at all levels of education.

## 9 Limitations of the study

The studies included in this research are limited to master's and doctoral theses conducted in Türkiye and published in the National Thesis Center. Graduate theses were preferred because a significant portion of experimental values education studies conducted in Türkiye are produced within the scope of graduate programs. Furthermore, the inclusion of unpublished theses aimed to reduce publication bias by minimizing overrepresentation of statistically significant findings. The study covers theses published until December 31, 2025. Only experimental research is included in the meta-analysis. Qualitative research, survey studies, or mixed-methods research are excluded. The study analyzes pre-school, primary, secondary, high school, and university levels separately. The study examines research conducted before and after the 2018 curriculum change separately. However, further studies are needed to fully assess the long-term effects of the curriculum changes. The studies were conducted in different regions and schools, but variables such as socioeconomic status, cultural differences, and school environment were not controlled for. These factors may be decisive in determining the impact of values education.

Finally, the methodological quality and risk of bias of the included studies were not formally assessed using a standardized appraisal tool. Although all included studies were master's theses and doctoral dissertations that had undergone institutional academic review and examination processes, the methodological information reported in many theses was insufficient to permit a consistent and reliable quality assessment. Therefore, the findings should be interpreted in light of this limitation. Future meta-analyses should incorporate formal methodological quality or risk-of-bias assessments when primary studies provide sufficient information.

## Data Availability

The raw data supporting the conclusions of this article will be made available by the authors, without undue reservation.
